# Ozone therapy improves early stages of osseointegration in ovariectomized rats

**DOI:** 10.1590/1678-7757-2023-0172

**Published:** 2024-03-14

**Authors:** William Phillip Pereira da SILVA, João Matheus Fonseca e SANTOS, Mônica Caroline de SOUZA, Stéfany BARBOSA, Anderson Maikon de Souza SANTOS, Edilson ERVOLINO, Ana Paula Farnezi BASSI, Cortino SUKOTJO, Leonardo P FAVERANI

**Affiliations:** 1 Universidade Estadual Paulista “Júlio de Mesquita Filho” Faculdade de Odontologia de Araçatuba Departamento de Diagnóstico e Cirurgia Araçatuba Brasil Universidade Estadual Paulista “Júlio de Mesquita Filho” (UNESP), Faculdade de Odontologia de Araçatuba, Departamento de Diagnóstico e Cirurgia, Araçatuba, Brasil.; 2 Universidade Estadual Paulista “Júlio de Mesquita Filho” Faculdade de Odontologia de Araçatuba Departamento de Ciências Básicas Araçatuba Brasil Universidade Estadual Paulista “Júlio de Mesquita Filho” (UNESP), Faculdade de Odontologia de Araçatuba, Departamento de Ciências Básicas, Araçatuba, Brasil.; 3 University of Illinois College of Dentistry Department of Restorative Dentistry Chicago USA University of Illinois, College of Dentistry, Department of Restorative Dentistry, Chicago, USA.

**Keywords:** Osteoporosis, Ozone, Dental Implants, Dentistry, Preclinical Drug Evaluation

## Abstract

**Objective:**

the aim of this study was to analyze the influence of ozone therapy (OZN) on peri-implant bone repair in critical bones by installing osseointegrated implants in the tibia of ovariectomized rats.

**Methodology:**

ovariectomy was performed on 30 Wistar rats, aged six months (Rattus novergicus), and, after 90 days, osseointegrated implants were installed in each tibial metaphysis. The study groups were divided into the animals that received intraperitoneal ozone at a concentration of 700 mcg/kg — OZ Group (n=15) — and a control group that received an intraperitoneal saline solution and, for this reason, was named the SAL group (n=15). The applications for both groups occurred during the immediate post-operative period on the 2nd, 4th, 6th, 8th, 10th, and 12th day post-surgery. At various stages (14, 42, and 60 days), the animals were euthanized, and tests were performed on their tibiae. These tests include histomorphometric and immunohistochemical analyses, computerized microtomography, sampling in light-cured resin for calcified sections, and confocal microscopy. The obtained data were then analyzed using One-way ANOVA and the Shapiro-Wilk, Kruskal-Wallis, and student t-tests (P<0.05).

**Results:**

our findings indicate that the OZ group (3.26±0.20 mm) showed better cellular organization and bone neoformation at 14 days (SAL group, 0.90±1.42 mm) (P=0.001). Immunohistochemistry revealed that osteocalcin labeling was moderate in the OZ group and mild in the SAL group at 14 and 42 days post-surgery. The data from the analysis of calcified tissues (microtomography, histometric, and bone dynamism analysis) at 60 days showed no statistically significant differences between the groups (P=0.32).

**Conclusion:**

it was concluded that ozone therapy anticipated the initial phases of the peri-implant bone repair process.

## Introduction

Bone tissue quality is an essential factor for osseointegration as the tissue needs to maintain its structural integrity to provide support and maintain mineral homeostasis during the dynamic process of bone resorption, formation, and mineralization.^1-3^ One of the greatest challenges for the success of functional rehabilitation by osseointegrated implants is the underlying systemic conditions in patients, such as the presence of osteoporosis and diabetes, which can reduce bone quality and density.^4-11^ The poor cellular metabolism of systemically compromised patients, i.e., their reduced ability to react at the cellular level in the face of metabolite challenges to which the body is constantly exposed, directly contributes to the failure of the treatment given that the implant/peri-implant bone tissue complex is subjected to constant thermal, chemical, and mechanical oscillations in a process of coupled remodeling.^12-14^

Ozone (O_3_) comprises an oxygen molecule with three atoms that can be produced for therapeutic use by a generator. This can be done by passing high voltage electrical discharges through gas, oil, or aqueous solutions.^15,16^ Studies that have evaluated the effect of ozone therapy on tissue repair have shown an improvement in blood circulation (microcirculation and peripheral circulation), tissue, and general metabolism. Due to its hemostatic repair properties, ozone acts by improving the oxygen transport capacity of blood, regulating antioxidant enzymes, and modulating immune cell responses.^17,18^ It creates a resistance against oxidative stress by stimulating the antioxidant system.^15^ In addition to its hemostatic repair properties, ozone therapy also possesses bactericidal, virucidal, fungicidal, analgesic, and detoxifying properties^18^. However, because of a lack of scientific evidence of these effects, the safe clinical use of ozone is yet to be implemented.

Studies using animal models have demonstrated that, as a novel therapeutic strategy, ozone therapy can stimulate and accelerate bone repair.^17-19^ Bone repair after tooth extraction and treatment of bisphosphonate-induced necrosis have demonstrated that ozone can stimulate cell proliferation and improve soft tissue healing,^20,21^ although further studies are needed to confirm these hypotheses. It is crucial to study treatments that optimize the continuous bone repair process in the implant/peri-implant bone tissue complex in individuals who tend to develop unfavorable systemic conditions, such as low bone density. Such research has become even more relevant due to increased life expectancies and easier access to rehabilitative treatments such as dental implants. The experimental animal model of osteoporosis induced by bilateral ovariectomy is a good option for this type of study since it leads to an important change in bone microarchitecture with a consequent reduction in bone mineral density, thus simulating a critical repair situation.^22,23^

This study aimed to analyze the influence of short-term ozone therapy on peri-implant bone repair in critical bones. Osseointegrated implants were introduced in the femurs of rats with low bone densities after ovariectomy. The null hypothesis was that the experimental groups would show no changes in peri-implant bone repair, regardless of the therapy used.

## Methodology

### Ethics approval and lab conditions

This study was approved by the Ethics Committee on Animal Use at Universidade Estadual Paulista (UNESP), School of Dentistry, Araçatuba, Brazil (#00431-2018). The procedures were performed in accordance with the Animal Arrive guidelines 2. For this study, 30 Wistar rats, aged six months (*Rattus novergicus*) and weighing 250–300 grams, were obtained from the Central Animal Facility at the Dentistry School of Araçatuba (FOA) – UNESP.

Throughout the experiment, the animals were kept in cages, each with up to four animals, in an environment with a stable temperature (22±2° C) and a controlled light cycle (12 hours light and 12 hours dark), with solid food (Ração Ativada Produtor^®^, Anderson & Clayton S.A. – Laboratório Abbot do Brasil Ltda, São Paulo, SP, Brazil) and water *ad libitum*, except during the 12 hours prior to surgery.

The number of samples was determined using the software SigmaPlot 12.0 “Sample Size for Anova” tool (San Jose California, USA) and considered data from a previous study^23^ as its primary outcome (histometric analysis): mean difference of 144.3, standard deviation of 41.13, and a power test of 95%, obtaining n=4 per group. A number of five samples was used due to the possibility of animal loss. The randomization of animals (groups OZ and SAL) was performed by the website https://www.random.org/.

All project participants have training and laboratory experience to develop and execute all stages of this study. However, all surgeries (installation of implants and euthanasia) were performed by a single qualified operator (WPPS) in order to standardize the employed techniques. The microscopic and microtomographic scanning images were acquired by an unblinded researcher due to the need for group identification. However, subsequent to obtaining all the images relevant to the analyses assessed in this study, data analyses were conducted by a blinded evaluator.

### Ovariectomy

All rats underwent bilateral ovariectomy according to the methods stated in the literature.^23-27^ After the administration of anesthesia in the form of xylazine hydrochloride (Xylazine - Coopers, Brazil, Ltda.) and ketamine hydrochloride (Ketamine, Fort Dodge, Animal Health Ltda.), the abdominal cavity was accessed. The ovaries and uterine horns were lacquered, and the ovaries were removed.^23,26^

### Implant installation

For this study, a total of 30 Ti-4Al-6V implants provided by Emfils Implantes Odontológicos (Itu, São Paulo, Brazil) were used. The implants were 2 mm in diameter and 4 mm in height with the surfaces treated according to parameters specified by the company (nitric acid, blasting with aluminum oxide, nitric acid). After fabrication, the implants were packaged and sterilized with gamma rays.

Then, 90 days after osteoporosis induction, all animals fasted for 12 hours prior to the surgical procedure. Next, they were sedated with a combination of 50 mg/kg of intramuscular ketamine (Vetaset - Fort Dodge Saúde Animal Ltda, Campinas, São Paulo, Brazil) and 5 mg/kg of xylazine hydrochloride (Dopaser - Laboratório Calier do Brasil Ltda - Osasco, São Paulo, Brazil). Following sedation, the tibial metaphysis was surgically accessed to install the implants. The milling sequence (drilling with a diameter of 1.4 mm) and installation of the implants using a digital key (1.2 mm) were performed in accordance with the method described in the literature.^23,26^ Each animal received a randomly distributed implant in just one of their tibias.

All implants were installed by the same trained surgeon, with two other researchers assisting them and performing sutures. All researchers involved in the surgical procedures were blinded to the experimental groups (SAL or OZ).

### Ozone therapy

It was decided to apply ozone in gaseous form to the peritoneum of the animals. The ozone was generated by a specific device (OZONE&LIFE INDÚSTRIA, COMÉRCIO E SISTEMAS LTDA, São José dos Campos - São Paulo) coupled to an oxygen cylinder receiving pure oxygen. It was subjected to a voltage of 5 - 13 mV, thus generating medical ozone, which was then collected in a syringe.

**Figure 1:**
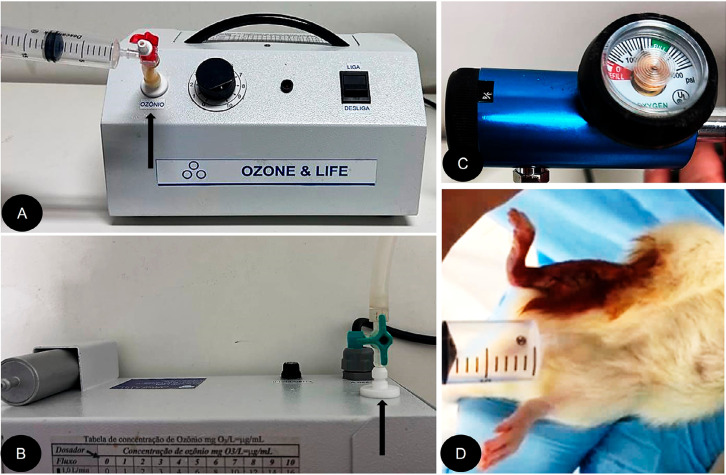
(A) Front view of the ozone generator while collecting 10ml with a 20-ml syringe. The ozonized gas outlet is indicated by the black arrow. (B) Top view of the ozone generator identifying the oxygen inlet location (black arrow). (C) Oxygen flow regulator set at 3/4 flow. (D) Intraperitoneal application of ozone during the immediate postoperative period

The application of ozone gas was performed via intraperitoneal injection. A concentration of 700 mcg/kg was administered using a 20 ml disposable syringe with a 25×0.07 mm needle. Considering the average weight of the animals, an oxygen flow of ¾ L/min was utilized, and the dosing instrument was set to a scale of 10. Each animal received 10 mL of ozone under these parameters (Figure 1).^28^

According to the application of ozone therapy or not, the animals were divided into two groups: 1) the OZ group (n=15), in which the concentration of ozone administered intraperitoneally was 700mcg/kg. The ozone was administered in the immediate postoperative (PO) period and on the 2nd, 4th, 6th, 8th, 10th, and 12th PO day.^21^ In the SAL group (n=15), an intraperitoneal administration of 1 ml of saline solution was performed using a 5-ml disposable syringe with a 25 mm×0.07 mm gauge needle in the immediate PO period on the 2nd, 4th, 6th, 8th, 10th, and 12^th^ day.

To analyze decalcified tissues, the animals were euthanized on days 14 and 42 after implant installation using an overdose of anesthesia (thiopental sodium, 150 mg/kg, intraperitoneally). The collected specimens were submitted for decalcified tissue analysis. In order to analyze calcified tissue, the animals were euthanized at 60 days after implant installation.

These intervals for euthanasia were chosen because they represent, in the chronology of bone repair in rats, an intermediate (14 days) and an advanced phase (42 days). Furthermore, the period of 60 days for calcified tissues was added in view of the proposal to apply fluorochrome calcein and alizarin red at 14 days and 42 PO days, and another 18 days up to 60 days for the analysis of calcified tissues due to bone dynamism (old bone/new bone). Additionally, it was assessed whether the effects of ozone therapy would be sustained after its suspension at 12 PO days.

## Peri-implant bone repair analysis

### Laboratory processing of decalcified tissue

#### Histological analysis

After the euthanasia of the animals on days 14 and 42 after implant installation, areas from the experimental surgery region (tibiae) were removed and five specimens from each group and period were subjected to decalcified histological processing. The tibiae with the installed implants were fixed in formalin and decalcified in EDTA (10%) for eight weeks. The implants were then removed by counterclockwise movement with a 1.2-mm hexagonal digital key. Next, the pieces were dehydrated in a sequence of alcohol at concentrations from 70 to 100%. After these steps, diaphanization with xylene and subsequent paraffin embedding were performed to obtain 5-μm thick sections mounted on histological slides. Following microtomy, the even-numbered slides were subjected to hematoxylin and eosin (HE) staining and the odd ones, to immunohistochemical analysis.

After staining, the slides were viewed and photographed under an optical microscope with a camera attached to it and subsequentially stored in TIFF format. The images were qualitatively analyzed with reference to the maturation of bone tissue and the relative amount of cells. Quantitative analysis was performed as described below.

#### Histometric analysis

The histological slides of the decalcified tissues were microphotographed, and the images were stored in TIFF files. Afterward, analysis was performed using Image J (Processing Software and Image Analysis, Ontario, Canada), delimiting the region to be measured with the “straight” tool. Next, the perimeter of bone formed at the bone/implant contact (BIC) was calculated in millimeters (mm). To calculate the area of new bone formation (NBF), the “free hands” tool was used to measure NBF in the region corresponding to the central spiral of the implant in mm^2^.^7^

#### Processing and immunohistochemical analysis

After the decalcification of the peri-implant tissues and preparation of the paraffin-embedded histological slides for immunohistochemistry reactions, endogenous peroxidase activity was inhibited with hydrogen peroxide. Next, the slides underwent the antigen retrieval step with citrate phosphate buffer (pH 6.0). The polyclonal primary antibody produced in goats, osteocalcin (OCN) (SC 18319; Santa Cruz Biotechnology, Dallas, TX, U.S.A.), was used for antigen retrieval to analyze cellular responses to the mineralization process. The biotinylated anti-rabbit secondary antibody produced in rabbits (Pierce Biotechnology) was used with avidin and biotin as amplifiers (PK 6100; Kit Elite, Vector Laboratories). Finally, diaminobenzidine (Dako, Denmark, SC, U.S.A.) was used as a chromogen.

After this step, counterstaining with Mayer’s hematoxylin was performed. For the used antibody, protein expression was semi-quantitatively evaluated by assigning different scores according to the number of immunolabeled cells in the peri-implant repair. The analysis was performed under an optical microscope (LeicaR DMLB, Heerbrugg, Switzerland) by an experienced histologist (E.E.). The number of cells marked by staining was recorded using the following scores: no staining (0); light staining (1 - up to 25% labeling cells), moderate staining (2 - up to 50% labeling cells), and intense staining (3 - more than 75% labeling cells), with diaminobenzidine staining being considered positive. Care was taken to perform the same experiments on the negative controls to evaluate the specificity of the antibodies.^29,30^ Immunostainings for osteocalcin were analyzed in connective tissue, targeting young osteoblasts and osteocytes in areas of the extracellular matrix.

## Laboratory processing of calcified tissue

### Computerized Micro-CT

For the three-dimensional structural analysis of bone tissue, five rats belonging to each group (OZ and SAL) had their tibiae removed after euthanasia at 60 PO days, reduced, and stored in 70% alcohol. First, the pieces were subjected to X-ray scanning analysis in a digital computerized micro-tomography system. The specimens were scanned with the use of a SkyScan microtomograph (SkyScan 1176 Bruker MicroCT, Aatselaar, Belgium, 2003), using 8-µm thick sections (90Kv and 111μA), a copper-aluminum filter, and a rotation step of 0.05 mm. The images obtained by projecting X-rays onto the samples were stored and reconstructed by determining the area of interest using the NRecon software (SkyScan, 2011; Version 1.6.6.0).

Employing the Data Viewer software (SkyScan, Version 1.4.4 64-bit), the images were reconstructed to fit the standard positioning for all samples, enabling the observation in three planes (transverse, longitudinal, and sagittal). Then, using CTAnalyser — CTAn software (2003-11SkyScan, 2012 Bruker MicroCT Version 1.12.4.0), a circular area was defined around the entire implant (ROI) and bounded by 0.25 mm around the entire implant. This area was defined as the Total Area (including the 0.25-mm margin around the implants - ROI 2.38mmx2.38mm). The CTAn software measures the image according to a grayscale (threshold). After adjusting and removing the shades of gray from the corresponding implant area, the threshold used in the analysis totaled 45-255 shades of gray, which made it possible to obtain the volume of bone formed around the implants.

Following the volumetric parameters suggested by the American Academy of Mineralized Bone Research, the parameters for bone tissue quantity (BV.TV= bone volume percentage) and bone tissue quality (Tb.Th= trabecular thickness, Tb.SP= separation of trabeculae, Tb.N= number of trabeculae, and To [tot] = total porosity percentage) were obtained.

## Confocal laser microscopy (peri-implant bone dynamics)

The same tibia specimens of the Micro-Ct were subjected to laboratory processing of the calcified tissues. The specimens were gradually dehydrated with increasing concentrations of alcohol at 70, 80, 90, 95, and 100%, with solution renewal every three days. The dehydrating pieces were placed in an orbital shaker (KLine CT - 150, Cientec - Equipamentos para Laboratório, Piracicaba, São Paulo, Brazil) for four hours every day. Then, the specimens were immersed in a mixture of 100% alcohol and light-curing resin Techno Vit^®^ (Germany, Heraeus Kulzer GmbH Division Technik Philipp-Reis-Str. 8/13 D-61273 Wehrheim). They were enclosed in Technovit resin and light-cured in accordance with the protocol for Exakt processing (Cutting System, Apparatebau, Gmbh, Hamburg, Germany). The samples were cut and ground using a cutting system and an automatic polishing machine (Exakt Cutting System, Apparatebau, Gmbh, Hamburg, Germany) until approximately 80-μm thick sections were obtained.

These sections were then mounted on slides and analyzed using a laser confocal microscope (Leica CTR 4000 CS SPE; Leica Microsystems, Heidelberg, Germany). The images had a size of 1×1 mm^2^ and corresponded to optical sections of 512×512 pixels. Sections of 2 μm were scanned for 1 hour and 30 minutes. Following this procedure, 28 sections were obtained for each 56-μm scan. The barrier filters used were BP 530/30 nm and 590 LP combined with “double dochroic” 488/568-nm activation and the photomultiplier was set at 534 for calcein and at 357 for alizarin. The images obtained by confocal microscopy were reconstructed using a software for confocal microscope manipulation (Leica CTR 4000 CS SPE, Leica Microsystems, Heidelberg, Germany).

The period of fluorochrome application was at 14 days, with calcein (green) representing the old bone and 42 days, with alizarin (red), the new bone. Fluorochromes are chemical components that can bind to calcium at the time of precipitation in the organic matrix, thus enabling the measurement of bone neoformation. The images were saved in TIFF format and analyzed on the ImageJ software (Processing Software and Image Analysis, Ontario, Canada) using the color threshold tool. The hue, saturation, and brightness of each image were standardized. First, the calcein in the sample was highlighted and the “measure” tool was used to provide the area in pixel^2^. The same procedure was performed for alizarin, obtaining data on the dynamics of the alveolar bone. Bone turnover was represented by the difference between old bone (green) and new bone (red).

### Histometry

Next, the specimens that were mounted on histological slides in order to analyze calcified tissues using confocal microscopy analysis were washed in deionized water and stained with alizarin red and Stevenel blue. The aim of histometric analysis was to calculate the linear and bilateral extent of bone-implant contact (BIC) between the newly formed bone tissue and the area of new bone formation (NBF) in the most central spiral on each side of the implant. The photomicrographs of the histological slides were saved in TIFF format and analyzed with the Image J software (Processing Software and Image Analysis, Ontario, Canada). Then, the perimeter of BIC was calculated in pixel^2^ using the “straight” tool. To obtain the NBF data, the “free hands” tool was used to measure the area of newly formed bone in the region corresponding to the most central spiral of the implant in pixel^2^.^7^

### Statistical analysis

All data obtained from the analyses were subjected to a normality test (Shapiro-Wilk) with a significance level of 5%. For all analyses, the software SigmaPlot 12.0 (Exakt Graphs and Data Analysis, San Jose, California, USA) was used with a 5% significance level. The data from BIC and NBF were subjected to one-way ANOVA. For microtomography (the BV.TV., Tb.Th, Tb.N, Tb.Sp, and Po.[tot]) parameters, the student’s t-test was applied. The Kruskal-Wallis test was performed on peri-implant bone dynamism (Calcein and Alizarin staining) and immunohistochemical data (OCN).

## Results

### Decalcified tissue analysis

#### Histological parameters (HE staining)

**Figure 2:**
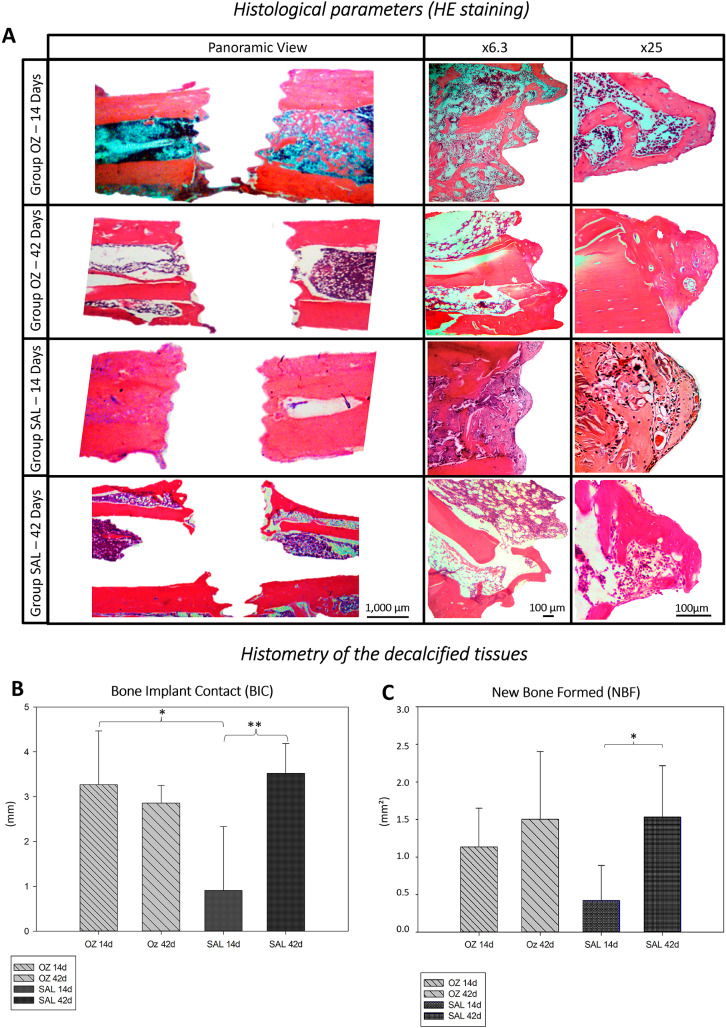
(A) Histological photomicrographs (HE) for OZ and SAL groups at 14 and 42 days. (B) Graphic image of the Bone Implant Contact (BIC) for the OZ and SAL groups at 14 and 42 days. * Statistical difference between OZ and SAL groups at 14 days (P=0.001); **Statistical difference between SAL 14 days and SAL 42 days (P=0.001). (C) Graphic image of the area of New Bone Formation (NBF) for the OZ and SAL groups at 14 and 42 days. * Statistical difference between the 14 and 42 day periods for the SAL group (P=0.005)

Histological photomicrographs (Figure 2-A) showed that, at 14 days, there was an increase in cells in the medullary canal, as well as an accelerated response in the organization of connective tissue, with the presence of areas of bone formation in the OZ group near the implant spires. Compared to the SAL group, at 14 days, the presence of organized connective tissue with few areas of bone formation near the implant threads was noted.

At 42 days, both groups (OZ and SAL) showed bone neoformation around the implant threads, with a decrease in the number of cells in the medullary canal of the tibiae, when compared to the 14-day period for both groups.

#### Histometry of the decalcified tissues

At 14 days, BIC in the medullary region for the OZ group was 3.26±0.20 mm, with a total of 84% bone formation in contact with the implants. For the same group, at 42 days, the average BIC was 2.85±0.396 mm, again with 84% bone formation in contact with the implants. The intragroup statistical analysis of the OZ group on days 14 and 42 revealed no statistically significant differences (*P*=0.50) (Figure 2- B).

In the linear extension analysis of BIC in the SAL group, at 14 days, a mean value of 0.908±1.42 mm was obtained, with 16% of the bone formation in contact with the implants. At 42 days, an average of 3.520±0.664 mm BIC was found, with 82% bone formation in contact with the implants. Hence, there was a statistically significant difference between the data from the 14 and 42-day periods in the SAL group (*P*=0.001).

The comparison between OZ and SAL at 14 days showed a statistical difference (*P*=0.001), whereas, at 42 days, there was no statistical difference (*P*=0.28). After 14 days, the OZ group had an NBF of 1.13±0.51 mm^2^, with a 51.02% of neoformed bone. After 42 days, this value had grown to 1.50±0.90 mm^2^, with a total of 71.42% neoformed bone. This difference (*P*= 0.34) was, however, statistically insignificant.

For the SAL group, on day 14, the area of bone formation was 0.420±0.46 (mm^2^), totaling 19.24% of a newly formed bone area. On day 42, results were 1.53±0.68 (mm^2^), totaling 65.79% of formed new bone. The SAL intragroup comparison between days 14 and 42 showed a statistically significant difference (*P*=0.005), whereas the intergroup comparison (OZ and SAL) showed no significant difference at 14 (*P*=0.051) or 42 days (*P*=0.93) (Figure 2-C).

#### Immunohistochemical analysis

**Figure 3:**
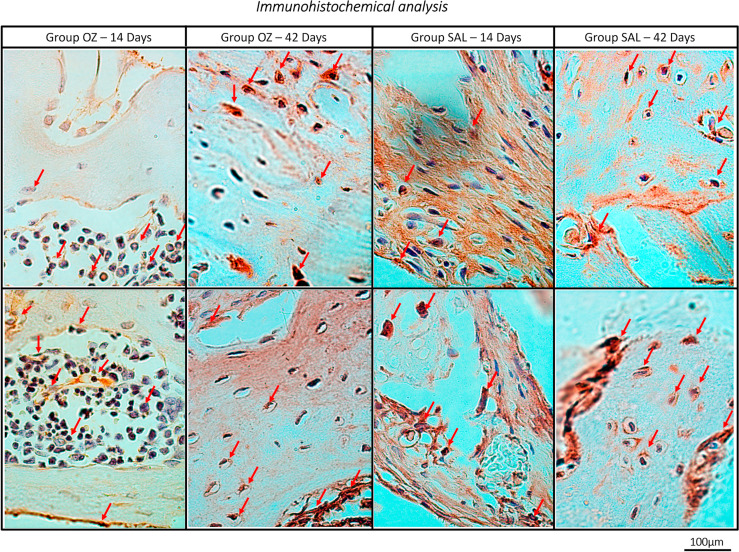
Photomicrographs of the experimental groups OZ and SAL representing the positive immunostaining for OCN protein. At 14 days - Group OZ score 2 (moderate labeling) and Group SAL score 1 (mild); At 42 days Group OZ and Group SAL score 2 (moderate labeling). Original magnifications at 40x. Immunolabeling: arrows in red

After 14 days, most slides pertaining to the OZ group showed more than half of the connective tissue with osteocalcin labeling, with an emphasis on osteoblasts in the peri-implant region, whereas the SAL group showed light labeling, with almost 25% osteoblasts (*P*<0.05). On day 42, both groups had stabilized, showing moderate OCN labeling (*P*>0.05) (Figure 3).

## Calcified tissue analysis

### Microtomographic analysis

**Figure 4:**
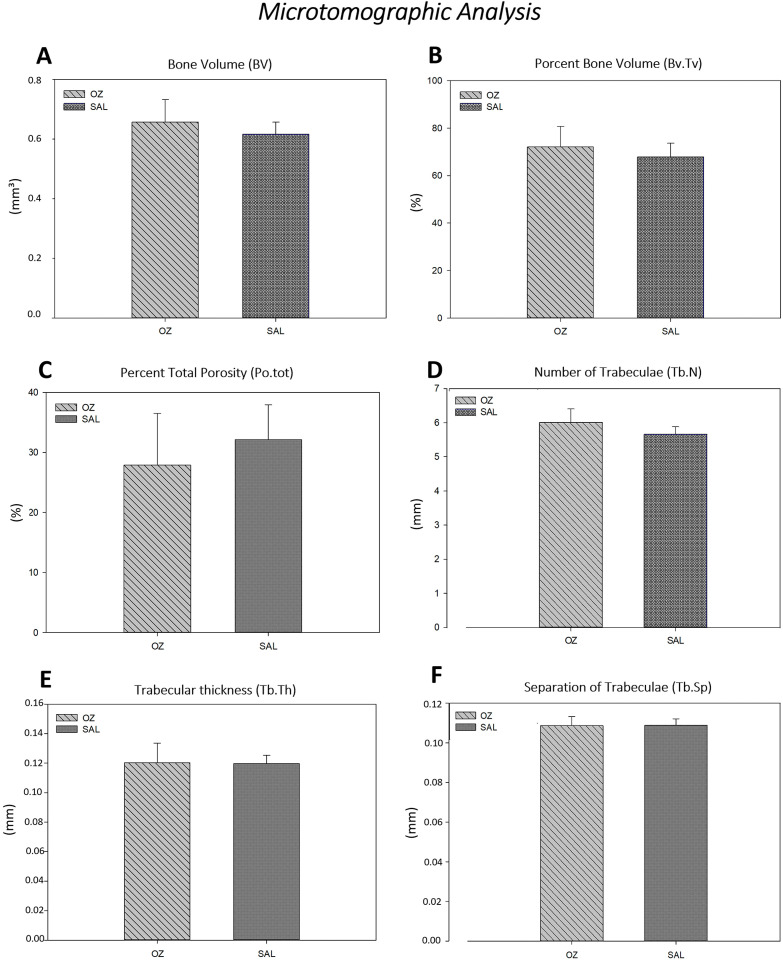
Graphic image of the quantitative parameters (A) - Bv; (B) - Bv.Tv; (C) - Po.tot and the qualitative parameters (D) - Tb.N; (E) - Tb.Th; (F) - Tb.Sp in the comparison of the tested groups (OZ and SAL) (P>0.05)

Comparative analysis of the quantitative (Bv; Bv.Tv; Po.tot) and qualitative parameters (Tb.N; Tb.Th; Tb.Sp) between the tested groups (OZ and SAL) revealed no statistically significant differences (*P*>0.05) (Figure 4).

The mean values for the bone volume parameter (Bv) were 0.65±0.07 /mm^3^ for the OZ group and 0.61±0.04 /mm^3^ for SAL. The mean values of bone volume percentages (Bv.Tv) were 72.12±8.58% for the OZ group and 67.89±5.79% for the SAL group. Furthermore, the percentage of total porosity (Po.tot) was 27.87±8.58% in the OZ group and 32.10±5.79% in the SAL group.

In the qualitative analysis, the mean values of the number of trabeculae (Tb.N) were 6.0±0.40 mm for the OZ group and 5.66±0.21 mm for the SAL group. The average trabecular thickness (Tb.Th) was 0.12±0.01 mm in the OZ group and 0.11±0.005 mm in the SAL group. Finally, the separation in millimeters between the trabeculae (Tb.Sp) was 0.108±0.004 mm in the OZ group and 0.108±0.003 mm in the SAL group.

## Confocal laser microscopy (peri-implant bone dynamics)

**Figure 5:**
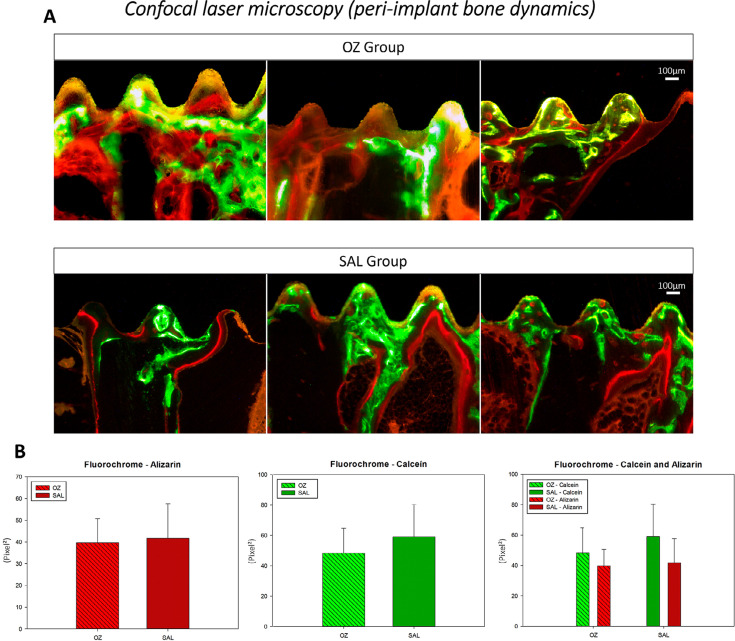
(A) Representative image of the fluorochromes for the OZ and SAL groups. Original magnifications at 10x (B) Representative graphical images of the fluorochromes

The analysis of the peri-implant bone by fluorochrome calcein staining showed mean values of 48.36±16.29 (pixel^2^) for the OZ group and of 59.12±21.25 (pixel^2^) for the SAL group. There was no statistically significant differences between groups with respect to calcein staining (*P*=0.452). Alizarin fluorochrome staining showed mean values of 39.71±10.976 (pixel^2^) in the OZ group and 41.72±15.86 (pixel^2^) in the SAL group. The comparative statistical analysis between the two groups showed no significant differences (*P*=0.84). Moreover, comparative analysis between the fluorochromes calcein and alizarin showed no significant differences between both groups (OZ *P*= 0.41; SAL *P*=0.23) (Figure 5 – A and B).

## Histometry (Calcified)

**Figure 6:**
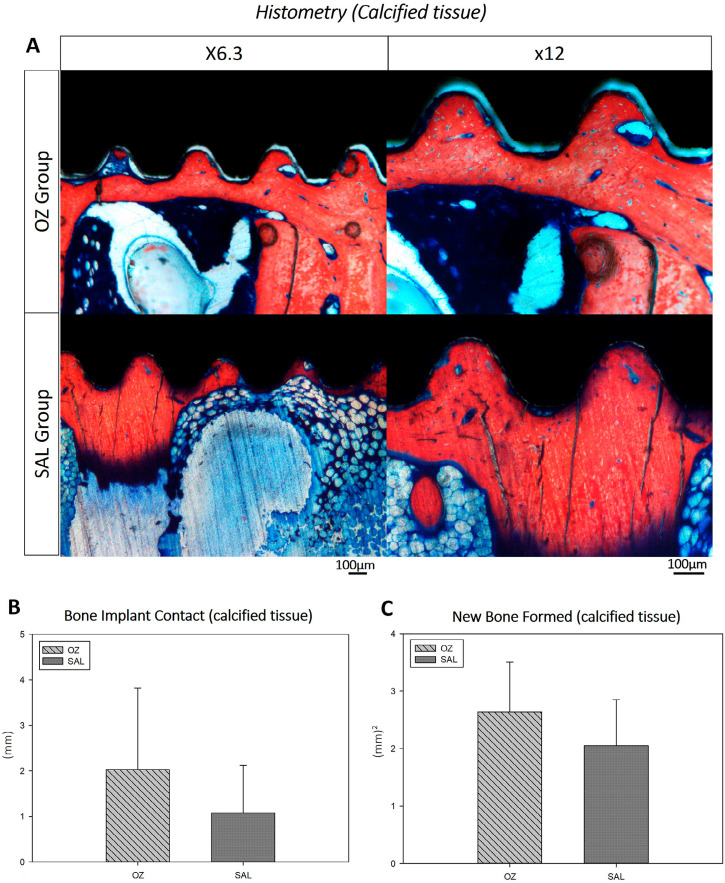
(A) Representative images for the histometric analysis of experimental groups OZ and SAL (alizarin red and Stevenel blue staining). (B) Graphical image of the analysis of bone implant contact between the OZ and SAL groups at 60 days (P=0.321). (C) Graphical image of the analysis of new bone formation area between groups (OZ and SAL) (P=0.763)

The mean values of the linear extension of BIC in the medullary region in millimeters observed in the OZ group at 60 days was 2,029±1,795 (mm), with a total of 41.38% bone formation in contact with the implant In the SAL group, the mean BIC length at 60 days was 1,078±1,042 (mm) with 26.96% bone formation in contact with the implant. In the comparison between OZ and SAL at 60 days, no statistically significant differences were noted (*P*=0.321) (Figure 6 – A and B).

In the analysis of bone neoformation, the mean values for the OZ group were 2.63+0.86 (mm^2^), totaling 76.83% of neoformed bone. For the SAL group, mean values were 2.05+0.800 (mm^2^), totaling 76.45% of neoformed bone. The comparative statistical analysis of NBF between the two groups, OZ and SAL, showed no statistical differences (*P*=0.763) (Figure 6-A and C).

## Discussion

Ozone therapy has been extensively researched and clinically applied in dentistry and medicine.^31-34^ However, few preclinical studies have evaluated the action of ozone on bone metabolism.^17,19^ In this study, ozone therapy was shown to contribute to cell proliferation and organization in the initial phases of bone repair in the peri-implant bone repair process.

The animal model proposed in this study is already well-established in the literature for evaluating effects on peri-implant repair.^26,36-38^ Ovariectomy induces a significant reduction in estrogen levels, simulating a condition similar to menopause and post-menopause in women.^9,10^ Hormonal suppression results in decreased osteoblast activity and an increase in bone reabsorption by osteoclasts, ultimately leading to reduced bone density and subsequent bone mineral loss.^13^ Studies such as Polo, et al.^23^ (2020) and Momesso, et al.^23^ (2020) have demonstrated that peri-implant bone repair in osteoporosis-induced (ovariectomized) rats is more critical than in healthy animals with normal bone density due to bone loss and increased osteoclastogenic activity.^23,26^

Osseointegration is directly influenced by bone quality. By creating a critical condition for bone repair, it becomes possible to conduct a more precise analysis of the effects of ozone therapy. This approach serves as a valuable proposal for osseointegration studies and therapies aimed at modulating bone biology. It is more feasible to assess cellular, mechanical-structural, and biological responses when bone undergoes alterations.

The quest for therapeutic strategies to mitigate the systemic changes affecting bone quality has seen remarkable growth, particularly in light of the global increase in life expectancy. In this context, approaches such as ozone therapy have gained prominence due to their ease of implementation, cost-effectiveness, and versatile applications. Ozone has a direct impact on the immune system and cellular metabolic responses, notably by stimulating the proliferation of immune-active cells and enhancing the synthesis of biologically active substances such as interleukins, leukotrienes, and prostaglandins.^39,40^ Furthermore, its role in promoting hypervascularization and subsequent tissue hyperoxygenation is crucial for effective bone repair.

Since very few studies have discussed the utilization of ozone in bone repair^16,18,21,35^, there is no consensus on the method of systemic administration, which can be performed in animals by intraperitoneal or rectal routes. Recently, there has also been consideration of topically applying ozone using oil vehicles. It has been noted that systemic application is associated with better outcomes in terms of bone formation and reduced bone loss.^41,42^ In this study, no complications resulted from intraperitoneal application in animals, demonstrating that it is a safe and easy administration technique.^21,43^

In this study, the parameters observed in its histological analysis (HE) at 14 days showed an increase in the number of cells in the region near the implants and in the tibial medullary canal, with better organization and the beginning of bone formation in the group that used ozone therapy (OZ). Conversely, the control group (SAL) showed a peri-implant repair process with a chronology consistent with what was previously noted in rats with decreased bone density caused by ovariectomy.

Although not a quantitative parameter, cellular organization is an important indicator of tissue repair, especially in earlier stages. In this context, the more organized the cell layers, the lower the number of fibroblasts, and the greater the number of blood vessels and osteoblasts, the more mature is the tissue. This information is consistent with the findings for the OZ group, which even showed the beginning of bone formation. These results corroborate studies that have demonstrated the optimizing influence of ozone therapy in the initial phases of bone repair.^18,19^

The findings of bone-implant contact (BIC) by the histometric analysis of decalcified tissues demonstrated that, at 14 days, in the OZ group, there was a greater interface between titanium and neoformed bone in the medullary canal of tibiae than in the SAL group. This finding is of great importance, especially when considering the primary stability of implants, which directly depends on the formation of a good bone interface in the first moments after implant installation. Furthermore, these results are consistent with findings from other authors who have demonstrated that ozone therapy serves as an optimizing agent for bone repair, employing the same dosage of 700 mcg/kg and similar application periods.^41,42^

At 42 days, both groups (OZ and SAL) showed bone neoformation around the spires of the implants and a decrease of cells in the medullary canal of the tibias, when compared to the 14th day of each group. These results were described in a study by Duman, et al.^35^ (2017), which demonstrated the influence of ozone therapy in the initial phase (14 days) and at 42 days postoperatively. The results with regard to bone formation and mineralization in the control group and the test group were similar.^35,26^

It is important to highlight that, given the findings of this preclinical study and due to the short useful life of this therapy, the effects of ozone on bone repair occurred especially in the initial periods of repair, up to 14 days, with its application up to 12 PO days. In itself, this effect is already of great relevance for the biology of peri-implant repair since it is in the immediate PO period that there is a greater demand for tissue hypervascularization. At that point, proteins, cytokines, and undifferentiated mesenchymal cells can reach the defect region and differentiate into osteoblasts to subsequently begin bone remodeling. When thinking mainly about conditions of reduced bone mineral density such as osteopenia and osteoporosis, having a therapy that provides conditions for the repair to get off to a good start would, even if indirectly, be related to therapeutic success. However, if new challenges arise in later periods of peri-implant repair, whether due to fluctuations in the patient’s systemic condition, mechanical changes related to immediate occlusal loads or even microbiological changes, the use of ozone therapy should be considered in cases such as peri-implantitis. This therapy could at a later stage serve as a compensatory mechanism for conditions of hypoxia, hypocellularity, and hypovascularization. Therefore, future studies that apply ozone at a later stage (42/60 days) are recommended.

Complementing the histological results, the analyses of calcified tissues at 60 days after implant installation, both groups (OZ and SAL) presented similar results in bone formation and mineralization. The analysis of bone turnover by fluorochromes corroborated the analyses of BIC and NBF and showed no significant differences. However, a tendency toward better bone organization in the medullary region of the implant installation was noted.

Tissue oxygenation plays a central role in bone formation because it stimulates growth factors (IGF-1, PDGF, TGF-, FGF, VEGF) and the proliferation and differentiation of osteoblasts.^18,35^ Immunohistochemical analysis has shown higher (moderate) OCN staining in the OZ group at 14 days than the control group (mild staining). Growth factors in the initial periods of bone repair (7 and 14 days) should be investigated in future studies for a better understanding of the performance of ozone therapy in bone repair.

Despite promising results in the initial repair periods, this study presented some limitations that are unable to fully evaluate the effects of ozone in later repair periods. Future preclinical studies should prolong therapy to verify whether ozone offers a continued contribution to the optimization of bone repair or whether there is even a deactivation of cells after a certain stage. There is also a direct need for clinical research since ozone is already used in clinical practice in the field of dentistry.^33,34,44,45^

## Conclusion

Based on this preclinical study, it can be concluded that the systemic administration of ozone anticipated the initial periods of peri-implant bone repair in ovariectomized rats. Furthermore, it was found that due to the short useful life of ozone molecules, their effects only occur while they are being applied. As such, future studies that investigate the peri-implant effects of ozone over longer time intervals are of great relevance.

## References

[1] Aghaloo T, Pi-Anfruns J, Moshaverinia A, Sim D, Grogan T, Hadaya D (2019). The effects of systemic diseases and medications on implant osseointegration: a systematic review. Int J Oral Maxillofac Implants.

[2] Branemark PI (1983). Osseointegration and its experimental background. J Prosthet Dent.

[3] Rosa AL, Crippa GE, Oliveira PT, Taba M, Lefebvre LP, Beloti MM (2009). Human alveolar bone cell proliferation, expression of osteoblastic phenotype, and matrix mineralization on porous titanium produced by powder metallurgy. Clin Oral Implants Res.

[4] Briot K, Geusens P, Em Bultink I, Lems WF, Roux C (2017). Inflammatory diseases and bone fragility. Osteoporos Int.

[5] Chandran S, John A (2019). Osseointegration of osteoporotic bone implants: role of stem cells, silica and strontium – a concise review. J Clin Orthop Trauma.

[6] Chrcanovic BR, Albrektsson T, Wennerberg A (2014). Diabetes and oral implant failure: a systematic review. J Dent Res.

[7] Faverani LP, Polo TO, Ramalho-Ferreira G, Momesso GA, Hassumi JS, Rossi AC (2018). Raloxifene but not alendronate can compensate the impaired osseointegration in osteoporotic rats. Clin Oral Investig.

[8] Grisa A, Veitz-Keenan A (2018). Is osteoporosis a risk factor for implant survival or failure?. Evid Based Dent.

[9] Lerner UH (2006). Bone remodeling in post-menopausal osteoporosis. J Dent Res.

[10] Lerner UH (2006). Inflammation-induced bone remodeling in periodontal disease and the influence of post-menopausal osteoporosis. J Dent Res.

[11] Manrique N, Pereira CC, Luvizuto ER, Sánchez MD, Okamoto T, Okamoto R (2015). Hypertension modifies OPG, RANK, and RANKL expression during the dental socket bone healing process in spontaneously hypertensive rats. Clin Oral Investig.

[12] Faverani LP, Silva WP, Sousa CA, Freitas G, Bassi AP, Shibli JA (2022). Mapping bone marrow cell response from senile female rats on Ca-P-doped titanium coating. Materials (Basel).

[13] Manolagas SC (1998). Cellular and molecular mechanisms of osteoporosis. Aging (Milano).

[14] Qadir A, Liang S, Wu Z, Chen Z, Hu L, Qian A (2020). Senile Osteoporosis: The Involvement of Differentiation and Senescence of Bone Marrow Stromal Cells. Int J Mol Sci.

[15] Souza AK, Colares RR, Souza AC (2021). The main uses of ozone therapy in diseases of large animals: a review. Res Vet Sci.

[16] Ozdemir H, Toker H, Balcı H, Ozer H (2013). Effect of ozone therapy on autogenous bone graft healing in calvarial defects: a histologic and histometric study in rats. J Periodontal Res.

[17] Gonencİ R, Tabur M, Ozsoy SY (2017). Preventive and curative effects of medical ozone in rats exposed to experimental osteomyelitis. Pak Vet J.

[18] Alpan AL, Toker H, Ozer H (2016). Ozone therapy enhances osseous healing in rats with diabetes with calvarial defects: a morphometric and immunohistochemical study. J Periodontol.

[19] Buyuk SK, Ramoglu SI, Sonmez MF (2016). The effect of different concentrations of topical ozone administration on bone formation in orthopedically expanded suture in rats. Eur J Orthod.

[20] Di Fede O, Del Gaizo C, Panzarella V, La Mantia G, Tozzo P, Di Grigoli A (2022). Ozone infiltration for osteonecrosis of the jaw therapy: a case series. J Clin Med.

[21] Erdemci F, Gunaydin Y, Sencimen M, Bassorgun I, Ozler M, Oter S (2014). Histomorphometric evaluation of the effect of systemic and topical ozone on alveolar bone healing following tooth extraction in rats. Int J Oral Maxillofac Surg.

[22] Momesso GA, Polo TO, Silva WP, Barbosa S, Freitas GP, Lopes HB (2021). Miniplates coated by plasma electrolytic oxidation improve bone healing of simulated femoral fractures on low bone mineral density rats. Mater Sci Eng C Mater Biol Appl.

[23] Polo TO, Silva WP, Momesso GA, Lima TJ, Barbosa S, Cordeiro JM (2020). Plasma electrolytic oxidation as a feasible surface treatment for biomedical applications: an in vivo study. Sci Rep.

[24] Ramalho-Ferreira G, Faverani LP, Grossi-Oliveira GA, Okamoto T, Okamoto R (2015). Alveolar bone dynamics in osteoporotic rats treated with raloxifene or alendronate: confocal microscopy analysis. J Biomed Opt.

[25] Chen J, Liu W, Zhao J, Sun C, Chen J, Hu K (2017). Gelatin microspheres containing calcitonin gene-related peptide or substance P repair bone defects in osteoporotic rabbits. Biotechnol Lett.

[26] Momesso GA, Santos AM, Santos JM, Cruz NC, Okamoto R, Barão VA (2020). Comparison between plasma electrolytic oxidation coating and sandblasted acid-etched surface treatment: histometric, tomographic, and expression levels of osteoclastogenic factors in osteoporotic rats. Materials (Basel).

[27] Ramalho-Ferreira G, Faverani LP, Momesso GA, Luvizuto ER, Puttini IO, Okamoto R (2017). Effect of antiresorptive drugs in the alveolar bone healing. A histometric and immunohistochemical study in ovariectomized rats. Clin Oral Investig.

[28] Shete AV, Subramaniam AV, Sable DM, Patil SV, Chavan MS, Shete MV (2016). Ozone therapy: healing properties of the blue gas. Int J Oral Health Dent.

[29] Manrique N, Pereira CC, Luvizuto ER, Rodriguez Sánchez MD, Okamoto T, Okamoto R (2015). Hypertension modifies OPG, RANK, and RANKL expression during the dental socket bone healing process in spontaneously hypertensive rats. Clin Oral Investig.

[30] Pedrosa WF, Okamoto R, Faria PE, Arnez MF, Xavier SP, Salata LA (2009). Immunohistochemical, tomographic and histological study on onlay bone graft remodeling. Part II: calvarial bone. Clin Oral Implants Res.

[31] Izadi M, Tahmasebi S, Pustokhina I, Yumashev AV, Lakzaei T, Alvanegh AG (2020). Changes in Th17 cells frequency and function after ozone therapy used to treat multiple sclerosis patients. Mult Scler Relat Disord.

[32] Tricarico G, Rodrigues Orlandin J, Rocchetti V, Ambrosio CE, Travagli V (2020). A critical evaluation of the use of ozone and its derivatives in dentistry. Eur Rev Med Pharmacol Sci.

[33] Uslu MÖ, Akgül S (2020). Evaluation of the effects of photobiomodulation therapy and ozone applications after gingivectomy and gingivoplasty on postoperative pain and patients’ oral health-related quality of life. Lasers Med Sci.

[34] Anzolin AP, Silveira-Kaross NL, Bertol CD (2020). Ozonated oil in wound healing: what has already been proven?. Med Gas Res.

[35] Duman IG, Davul S, Gokce H, Gonenci R, Özden R, Uruc V (2017). Effects of gaseous ozone treatment on bone regeneration in femoral defect model in rats. J Hard Tissue Biol.

[36] Polo TO, Momesso GA, Silva WP, Santos AM, Fonseca-Santos JM, Cruz NC (2021). Is an anodizing coating associated to the photobiomodulation able to optimize bone healing in ovariectomized animal model?. J Photochem Photobiol B..

[37] Luvizuto ER, Queiroz TP, Dias SM, Okamoto T, Dornelles RC, Garcia IR (2010). Histomorphometric analysis and immunolocalization of RANKL and OPG during the alveolar healing process in female ovariectomized rats treated with oestrogen or raloxifene. Arch Oral Biol.

[38] Dvorak G, Fügl A, Watzek G, Tangl S, Pokorny P, Gruber R (2012). Impact of dietary vitamin D on osseointegration in the ovariectomized rat. Clin Oral Implants Res.

[39] Alpan AL, Toker H, Ozer H (2016). Ozone therapy enhances osseous healing in rats with diabetes with calvarial defects: a morphometric and immunohistochemical study. J Periodontol.

[40] Swarna Meenakshi P, Rajasekar A (2022). A review on ozone therapy in periodontitis. Bioinformation.

[41] Erdemci F, Gunaydin Y, Sencimen M, Bassorgun I, Ozler M, Oter S (2014). Histomorphometric evaluation of the effect of systemic and topical ozone on alveolar bone healing following tooth extraction in rats. Int J Oral Maxillofac Surg.

[42] Saglam E, Alinca SB, Celik TZ, Hacisalihoglu UP, Dogan MA (2019). Evaluation of the effect of topical and systemic ozone application in periodontitis: an experimental study in rats. J Appl Oral Sci.

[43] Saglam E, Alinca SB, Celik TZ, Hacisalihoglu UP, Dogan MA (2019). Evaluation of the effect of topical and systemic ozone application in periodontitis: an experimental study in rats. J Appl Oral Sci.

[44] Sen S, Sen S (2020). Ozone therapy a new vista in dentistry: integrated review. Med Gas Res.

[45] Uraz A, Karaduman B, Isler SÇ, Gönen S, Çetiner D (2019). Ozone application as adjunctive therapy in chronic periodontitis: Clinical, microbiological and biochemical aspects. J Dent Sci.

